# The Fever of Neighboring Counties

**Published:** 1879-10

**Authors:** 


### THE FEVER OF NEIGHBORING COUNTIES.

It becomes our duty to chronicle the prevailing fever of certain sections of country contiguous to Atlanta. Exaggerated reports have been circulated through the secular press, and very incorrect views as to the nature of the prevailing affection. From these the idea of disease unknown to the medical profession is promulgated, producing quite a sensation in many parts of the country. In Polk, Dallas, Cobb and Cherokee, lying west of Atlanta, fever of a very fatal character has prevailed in certain neighborhoods. In some of these counties, fifty per cent of the cases have been reported to have proved fatal. The exaggeration to which we have above referred, consists principally in the number of persons attacked with the disease. If full credence be given to newspaper reports, we will believe that some neighborhoods have been almost depopulated, which is by no means true. The mortality has been indeed alarming, and most communities will fail to maintain perfect equilibrium under such fatality in their midst; and hence the sensational and exaggerated reports that have been circulated. The disease has not been confined to the counties mentioned. The lower part of Fulton and portions of DeKalb and Clayton have been visited by the same kind of fever, doubtless, but in a milder degree. While the same general character attended it south of Atlanta, malignancy excepted, perhaps not ten per cent. of the cases proved fatal.

It may appear strange to those who believe filth accumulations in densely populated cities generates malignant fevers, that the open, high and dry country surrounding Atlanta, in almost every direction, has been the subject of this fever, while it is rarely, if at all, found in the city.

We witnessed a similar fever in 1847 when the whole face of the earth uncultivated was prolific of mushrooms. The same state of things existed in July and August of the present year, and our prediction was that fever would be the result.

We propose at an early day to publish an article in the Journal, giving a succinct statement of our observations connected with this form of fever. Its duration, cause, and other peculiarities, make it differ from typhoid and other types.

